# Distinct Long‐Range Inhibitory and Excitatory Projections From the Mouse Superior Colliculus

**DOI:** 10.1002/cne.70216

**Published:** 2026-11-05

**Authors:** Kara K. Cover, Lupeng Wang, Ronald Paletzki, Charles R. Gerfen, Richard J. Krauzlis

**Affiliations:** ^1^ Laboratory of Sensorimotor Research National Eye Institute Bethesda Maryland USA; ^2^ Section on Neuroanatomy National Institute of Mental Health Bethesda Maryland USA

**Keywords:** brainstem, efferent connectivity, neurotransmitter identity, subcortical circuits, target specificity, thalamus, topographic organization

## Abstract

The superior colliculus (SC) is extensively connected with cortical and subcortical nuclei to facilitate a range of cognitive and sensorimotor functions. Efferent projections from the SC vary by originating layer and topographic location. The present study identifies an additional determinant of efferent connectivity: released neurotransmitter. We show that long‐range GABAergic and glutamatergic projection neurons produce distinct connectivity patterns in the mouse diencephalon, midbrain, and hindbrain. Targets strongly innervated by inhibitory vGAT+ neurons included the lateral dorsal, reuniens, anterior pretectal, and red nuclei, as well as the inferior colliculus. Targets of weaker vGAT+ innervation included the lateral habenula, ventral anterior–lateral complex, and reticular nucleus. In contrast, strongly innervated targets of excitatory vGlut2+ projections included the ventral medial, parafascicular, and tegmental reticular nuclei, whereas weaker innervation was present in the central medial intralaminar nucleus as well as in several contralateral thalamic and hindbrain nuclei. In shared efferent targets, vGAT+ and vGlut2+ projections often innervated different subregions or differed in cross‐hemispheric connections, notably in the zona incerta, periaqueductal gray, and lateral posterior, pedunculopontine, pontine reticular, and tegmental reticular nuclei. These results collectively demonstrate an additional dimension of complexity in SC efferent connectivity in mice. Inhibitory projections may facilitate SC‐mediated functions through a number of mechanisms independently of, or in concert with, excitatory projections. Future work examining the functional roles of SC long‐range inhibition stands to elucidate these mechanisms and their contributions to behavior.

## Graphical abstract

Transgenic mouse lines facilitated viral tracing of superior colliculus inhibitory (vGAT+; orange) and excitatory (vGlut2+; blue) neuronal projections throughout the brain. Example efferent targets are shown for diencephalon and midbrain coronal sections. Innervation patterns of these two populations differed in targeted nuclei, axonal density, and cross‐hemispheric projections. BioRender was used to create some of the graphics.

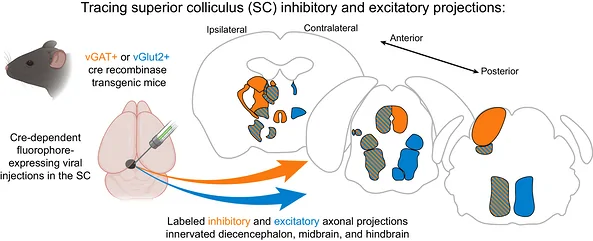

## 1 Introduction

The superior colliculus (SC) is an evolutionarily conserved structure in the mammalian midbrain that facilitates multimodal sensorimotor transformations and cognitive functions including attention and decision‐making (Basso et al. 2021; Krauzlis et al. 2013). This laminated nucleus is composed of superficial, intermediate, and deep layers. The superficial layers receive topographically organized visual signals from retinal ganglion cells and the visual cortex. In rodents, the superficial SC is the primary ascending route for visual information from the retina to the rest of the brain (May 2006). The intermediate and deep SC layers contain neurons that respond to multimodal sensory stimuli and are involved in initiating motor commands. In accordance with a role for this nucleus in sensorimotor function, specific projections from the rodent SC are associated with orienting (Cregg et al. 2020; Masullo et al. 2019; Solié et al. 2022), prey capture (Shang et al. 2019), and defensive responses to threat (Li et al. 2023; Wei et al. 2015; Zingg et al. 2017).

The diversity of SC‐mediated behaviors suggests that this region is anatomically connected with a multitude of functional networks. Indeed, the SC extensively innervates the diencephalon, midbrain, and hindbrain (Liu et al. 2022). Studies of SC connectivity show that efferent targets differ by SC layer (May 2006) and location in the retinotopic map (Benavidez et al. 2021). SC projection neurons are not exclusively glutamatergic, however. Specific long‐range GABAergic projections arising from the superficial layers have been identified (Choi et al. 2023; Gale and Murphy 2014; Li et al. 2023; Zhang et al. 2019), although these studies pertained exclusively to projections arising from the superficial SC and/or characterized circuits to specific efferents. Collectively, these previous studies suggest that long‐range inhibitory transmission may be a common mode of SC efferent connectivity, but an inclusive characterization of SC inhibitory projections has not been conducted. Understanding the manner of the SC's influence on efferent targets is essential to parsing the neural circuits that underlie this structure's contribution to sensorimotor and cognitive functions.

The goal of the present study was to characterize all inhibitory efferent connections of the SC and directly compare them to excitatory SC projections. Using transgenic reporter lines, we virally labeled inhibitory and excitatory neurons in the SC and examined innervation throughout the mouse brain. We observed distinct patterns of efferent connectivity between GABAergic and glutamatergic projection neurons. For efferents targeted by both cell types, we found that inhibitory and excitatory projections often innervated different subregions. These results elucidate an additional facet of SC efferent connectivity that can inform future work delineating the pathway‐specific functional contributions of the SC to sensory‐motor behavior.

## 2 Materials and Methods

### 2.1 Animals

vGAT‐Cre (strain #028862) and vGlut2‐Cre (strain #028863) mice bred on a C57BL/6J background were obtained from The Jackson Laboratory (Bar Harbor, ME, USA). These transgenic lines have been demonstrated to express Cre with high specificity in SC inhibitory and excitatory populations, respectively (Chen et al. 2025). Male and female mice aged 12–42 weeks were used in the study. Mice were housed with littermates (two to five per cage) in a 12‐h dark/light cycle and had ad libitum access to food and water. All experimental and animal husbandry procedures were approved by the NIH Institutional Animal Care and Use Committee (IACUC) and complied with the Public Health Service policy on the humane care and use of laboratory animals.

### 2.2 Surgical Procedures

Mice were anesthetized with isoflurane (4% induction, 0.8%–1.5% maintenance) and secured by a stereotaxic frame with ear bars. A feedback‐controlled heating pad was used to maintain the body temperature at 37°C, and ophthalmic ointment was applied to the eyes. Dexamethasone (1.6 mg/kg, s.c.) and meloxicam (2.0 mg/kg, s.c.) were administered to reduce inflammation and discomfort, respectively. Viruses were infused at a rate of 2 nL/s (50 nL/min maximum) using a microinjector (Nanoject III, Drummond Scientific, Broomall, PA, USA).

To selectively label Cre recombinase‐expressing SC neurons, AAV2‐Ef1a‐DIO‐hChR2‐mCherry or AAV2‐Ef1a‐DIO‐hChR2‐eYFP (UNC Vector Core, Chapel Hill, NC, USA) was unilaterally injected into the SC (100–200 nL; distance from bregma in mm, [AP] −3.86 to −4.15, [ML] 1.00 to 1.26, [DV] −1.75 to −2.10 in vGAT‐Cre (*N* = 5) or vGlut2‐Cre (*N* = 6) mice. To label both vGAT‐Cre expressing and putative excitatory neurons (i.e., cells not expressing Cre recombinase) in the same mice, AAV2‐Syn‐FLEX‐ChrimsonR‐tdTomato (UNC Vector Core) and AAV2‐Ef1a‐FLEX/OFF‐hChR2‐eYFP (Vector Biolabs, Malvern, PA, USA) were unilaterally injected into the SC (1:1 mix, 150 nL total; same coordinates as above) of vGAT‐Cre mice (*N* = 3).

### 2.3 Tissue Preparation and Immunohistochemistry

Four weeks after virus injections, mice were deeply anesthetized with isoflurane prior to transcardial perfusion with room‐temperature PBS (0.1 M, pH 7.35), followed by ice‐cold 4% paraformaldehyde in PBS. Brains were extracted and stored in 4% paraformaldehyde at 4°C. Fifty‐micrometer coronal sections were cut using either a Campden Instruments Vibrating Microtome (Lafayette, IN, USA) at room temperature or, following transfer to 20% sucrose PBS solution, a Leica freezing microtome (Leica Biosystems, Deer Park, IL, USA). Free‐floating sections were processed for immunohistochemical enhancement of fluorophore expression. Table 1 lists the primary and secondary antibodies used to enhance eYFP, mCherry, or tdTomato expression. Blue fluorescent Nissl stain (Neurotrace, N21479, Invitrogen, Carlsbad, CA, USA) was also applied to the tissue sections.

**TABLE 1:** Antibodies used for tracer fluorophore signal amplification.

Antigen	Manufacturer & catalog #	Dilution	Host species, isotype, clonality	RRID
RFP	Rockland Immunochemicals; 600‐401‐379	1:2000	Rabbit polyclonal IgG	AB_2209751
GFP	ThermoFisher; A10262	1:2000	Chicken polyclonal IgY	AB_2534023
Rabbit (AF555 conjugate)	ThermoFisher; A32732	1:200	Goat polyclonal IgG	AB_2633281
Chicken (AF488 conjugate)	ThermoFisher; A32931	1:200	Goat polyclonal IgY	AB_2762843

### 2.4 Histological Analysis

Following immunohistochemical processing, a subset of the cases was serially reconstructed and registered to a standardized atlas reference space (*N* = 2 vGlut‐Cre; *N* = 2 vGAT‐Cre; *N* = 3 FLEX on/off vGAT‐Cre mice). Every section from these brains was mounted on slides and imaged with a Zeiss ImagerM2 microscope configured with a motorized stage and camera controlled by Neurolucida imaging software (MBF Biosciences, Williston, VT, USA). The imaged serial sections were processed with NeuroInfo software (MBF Biosciences) to reconstruct a whole‐brain image volume that was registered to the Allen CCF mouse brain atlas to delineate the brain areas in which labeled neurons and axon processes were distributed.

Quantification of labeled cells and axons was performed using the NeuroInfo cell detection pipeline. Distribution of transfected cells is shown for each case that was processed through NeuroInfo (Figure 1; Table 2). Efferent targets of SC projections that were consistently observed across all cases are reported in Section 3 and summarized in Figure 14. Innervation strength was classified as either “strong” (dense innervation of either the entire nucleus or a confined subregion) or “weak” (diffuse input to either the entire nucleus or a subregion and consistently observed across cases). Quantification of axonal labeling from all cases processed in NeuroInfo is shown in Figures 12 and 13. Here, the axon terminal density for each efferent region was calculated by dividing the number of axonal boutons by the volume of the region (counts per cubic meter). A color gradient was applied, normalized to the maximum efferent innervation density within each case. All anatomical abbreviations are described in Table 3. References to atlas coordinates in the figures are based on the Franklin and Paxinos atlas (2007).

**FIGURE 1:**
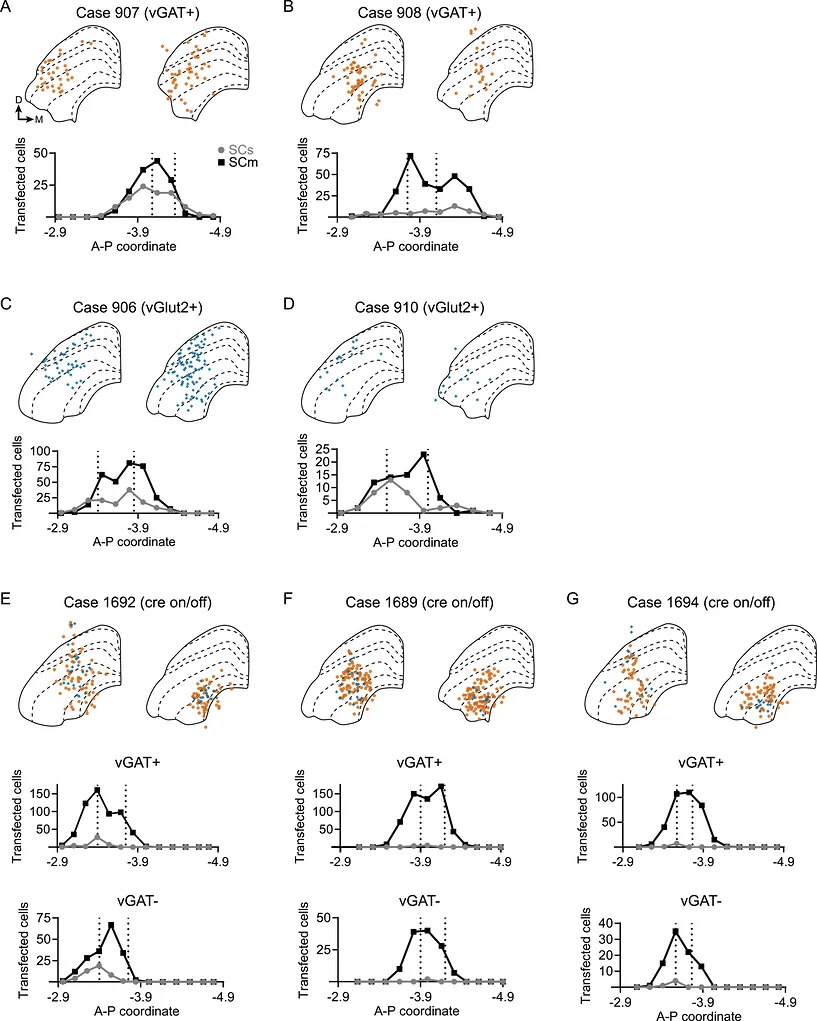
Superior colliculus cell labeling in NeuroInfo‐registered cases. (A) Top: Locations of virally transfected vGAT+ cells (orange circles) in two representative sections from a vGAT‐Cre mouse. Dashed lines denote the layers of the superior colliculus (SC). Bottom: SC cell counts summed across two adjacent sections, with coordinates relative to bregma. Dashed lines indicate the locations of example sections shown above. Cell counts are separately shown for superficial (gray) and intermediate and deep (black) layers. (B) Same as A, but for a second vGAT‐Cre case. (C, D) Same as A, but for vGlut2‐Cre mice with virally transfected vGlut2+ cells (blue diamonds). (E, F) Same as A, but for vGAT‐Cre mice injected with a dual‐fluorophore Cre on/off virus. Orange circles denote vGAT+ transfected cells, and blue diamonds indicate vGAT− transfected cells. See also Table 2. SCs, sensory‐related SC; SCm, motor‐related SC.

**TABLE 2:** Location of transfected cells (number; percentage of total).

	SCs	SCm	MRN	PAG	NOT	RSP	POST
*vGAT+*							
907	97; 37.2%	138; 52.9%	0; 0.0%	2; 0.8%	0; 0.0%	23; 8.8%	1; 0.0%
908	52; 15.7%	263; 79.2%	0; 0.0%	5; 1.5%	6; 1.8%	6; 1.8%	0; 0.0%
1692	41; 6.3%	560; 86.2%	0; 0.0%	31; 4.8%	0; 0.0%	18; 2.8%	0; 0.0%
1689	8; 1.3%	587; 92.9%	4; 0.6%	33; 5.2%	0; 0.0%	0; 0.0%	0; 0.0%
1694	8; 2.0%	363; 91.9%	11; 2.8%	10; 2.5%	0; 0.0%	3; 0.8%	0; 0.0%
*vGlut2+*							
906	134; 27.7%	318; 65.8%	2; 0.4%	7; 1.4%	3; 0.6%	10; 2.1%	9; 1.9%
910	38; 31.1%	73; 59.8%	1; 0.8%	1; 0.8%	1; 0.8%	5; 4.1%	3; 2.5%
*vGAT−*							
1692	45; 19.5%	180; 77.9%	1; 0.4%	1; 0.4%	0; 0.0%	4; 1.7%	0; 0.0%
1689	2; 1.6%	124; 97.6%	0; 0.0%	1; 0.8%	0; 0.0%	0; 0.0%	0; 0.0%
1694	5; 5.2%	86; 89.6%	0; 0.0%	2; 2.1%	0; 0.0%	3; 3.1%	0; 0.0%

**TABLE 3:** List of abbreviations.

	Abbreviations
3V	Third ventricle
A	Anterior
AP	Anterior–posterior
AH	Anterior hypothalamic nucleus
APN	Anterior pretectal nucleus
Aq	Cerebral aqueduct
AV	Anteroventral nucleus
CL	Central lateral intralaminar nucleus
CLR	Central linear nucleus of the raphe
CM	Central medial intralaminar nucleus
cpd	Cerebral peduncle
Cun	Cuneiform nucleus
D	Dorsal
dLGN	Dorsal lateral geniculate nucleus
DR	Dorsal nucleus raphe
dSCg	Deep superior colliculus, gray layer
dSCw	Deep superior colliculus, white layer
DV	Dorsal‐ventral
Eth	Ethmoid nucleus
fr	Fasciculus retroflexus
Hb	Habenula
IAD	Interanterodorsal nucleus
IAM	Interanteromedial nucleus
IC	Inferior colliculus
IFR	Interfascicular nucleus raphe
iSCg	Intermediate superior colliculus, gray layer
iSCw	Intermediate superior colliculus, white layer
L	Lateral
LD	Lateral dorsal nucleus
LDT	Laterodorsal tegmental nucleus
LH	Lateral habenula
LHA	Lateral hypothalamic area
LP	Lateral posterior nucleus
M	Medial
MD	Mediodorsal nucleus
ml	Medial lemniscus
ML	Medial‐lateral
mlf	Medial longitudinal fasciculus
MRN	Midbrain reticular nucleus
NB	Nucleus of the brachium of the inferior colliculus
NLL	Nucleus of the lateral lemniscus
NOT	Nucleus of the optic tract
NPC	Nucleus of the posterior commissure
NTB	Nucleus of the trapezoid body
OP	Olivary pretectal nucleus
opt	Optic tract
P	Posterior
PAG	Periaqueductal gray
PBG	Parabigeminal nucleus
PC	Paracentral intralaminar nucleus
Pf	Parafascicular intralaminar nucleus
PG	Pontine gray
PH	Posterior hypothalamic nucleus
PIL	Posterior intralaminar nucleus
PO	Posterior complex
PoL	Posterior limiting nucleus
POST	Postsubiculum
PoT	Posterior triangular nucleus
PP	Peripeduncular nucleus
PPN	Pedunculopontine nucleus
PPT	Posterior pretectal nucleus
PR	Perireunensis nucleus
PRC	Precommissural nucleus
PRNc	Pontine reticular nucleus, caudal part
PRNr	Pontine reticular nucleus, rostral part
PVH	Paraventricular hypothalamic nucleus
Re	Nucleus of the reuniens
Rh	Rhomboid nucleus
RM	Nucleus raphe magnus
RN	Red nucleus
RPF	Retroparafascicular nucleus
RR	Retrorubral area
RSP	Retrosplenial cortex
rst	Reticulospinal tract
RT	Reticular nucleus
rust	Rubrospinal tract
SAG	Nucleus sagulum
SCm	Superior colliculus, motor related
SCR	Superior central nucleus raphe
SCs	Superior colliculus, sensory related
SGN	Suprageniculate nucleus
SNc	Substantia nigra, pars compacta
SNr	Substantia nigra, pars reticulata
SPA	Subparafascicular area
SPF	Subparafascicular nucleus
SPFp	Subparafascicular nucleus, parvicellular part
sSCg	Superficial superior colliculus, gray layer
sSCo	Superficial superior colliculus, optic layer
sSCz	Superficial superior colliculus, zonal layer
STN	Subthalamic nucleus
TRN	Tegmental reticular nucleus
VAL	Ventral anterior‐lateral complex
vLGN	Ventral lateral geniculate nucleus
VM	Ventral medial nucleus
VMH	Ventromedial hypothalamic nucleus
VP	Ventral posterior complex
VPL	Ventral posterolateral nucleus
ZI	Zona incerta

## 3 Results

The goal of the present study was to characterize and compare the inhibitory and excitatory efferent connections of the SC. To that end, we virally labeled GABAergic or glutamatergic SC neurons using anterograde Cre‐dependent fluorophore‐expressing viruses and examined fluorescent axonal expression throughout the diencephalon, midbrain, and hindbrain of Cre reporter mice. In the following, we describe our findings separately for SC inhibitory and excitatory projections, before directly contrasting the two.

### 3.1 SC Inhibitory Projections

We used the vGAT‐Cre transgenic mouse to label inhibitory SC neurons, as this molecular marker more inclusively labels cells across all layers of the SC (Figure 2A) (Allen Institute for Brain Science 2025a) as compared to the commonly used GAD2 transgenic line, which preferentially labels superficial layer GABAergic cells (Allen Institute for Brain Science 2025b). We registered coronal brain sections to standardized atlas coordinates to provide agnostic classification of labeled SC vGAT+ axons throughout the brain.

**FIGURE 2:**
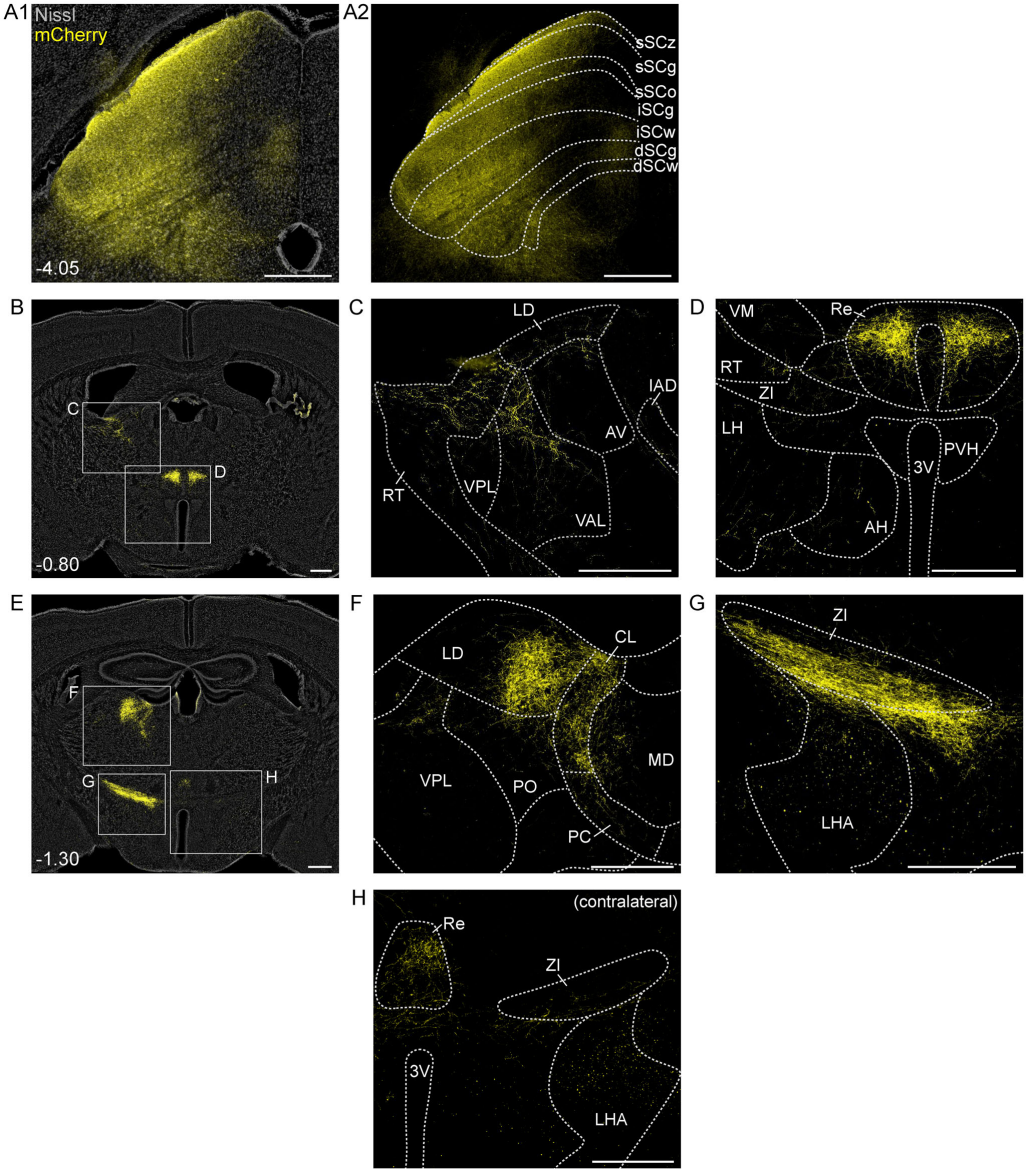
Mouse superior colliculus (SC) inhibitory neurons innervate the anterior diencephalon. (A) vGAT+ cells expressing mCherry fluorophore (yellow) in the SC of a representative case (#907) following unilateral injection of a Cre‐dependent anterograde virus in the SC of vGAT‐Cre transgenic mice. Inset coordinate indicates AP location relative to bregma. (B–H) SC vGAT+ axonal innervation of thalamic and hypothalamic regions. Micrographs in the left column depict the coronal section with Nissl (gray) counterstain. The center and right columns show magnified regions of interest. Anatomical abbreviations are detailed in Table 3. Scale bars = 500 µm.

As illustrated by a representative case, we observed SC vGAT+ inhibitory projections to many nuclei in the rostral diencephalon (Figure 2). In the thalamus, VGAT+ axons strongly innervated the nucleus reuniens bilaterally (Figure 2B,D) as well as the lateral dorsal and central lateral nuclei ipsilaterally (Figure 2E,F). Thalamic targets of weak or diffuse GABAergic innervation included the ipsilateral reticular nucleus (Figure 2B,C), ventral anterior–lateral nucleus (Figure 2B,C), and ventral medial nucleus (Figure 2B,D), as well as the ventral posterior complex, lateral mediodorsal nucleus, and paracentral nucleus (Figure 2E,F). SC GABAergic neurons sent a strong projection to the zona incerta ipsilaterally and a weaker projection contralaterally (Figure 2E,G,H). SC vGAT+ projections diffusely innervated other ipsilateral anterior diencephalic structures, including the anterior hypothalamic (Figure 2B,D) and lateral hypothalamic (Figure 2E,G) nuclei.

SC vGAT+ inhibitory projections also innervated many regions in the caudal diencephalon (Figure 3). vGAT+ neurons strongly innervated the ethmoid (Figure 3A,B), dorsal lateral geniculate (Figure 3A,B), and lateral posterior (Figure 3D,E) thalamic nuclei. We also observed weaker innervation of the parafascicular nucleus, posterior complex, and ventral lateral geniculate nucleus (Figure 3A,B), as well as the subparafascicular thalamic nucleus (Figure 3A–C). Diffusely targeted nuclei in the ipsilateral caudal thalamus included the posterior limiting, posterior triangular, posterior intralaminar, suprageniculate, and peripeduncular nuclei (Figure 3D,E). Strong ipsilateral innervation was observed in pretectal nuclei, including the nucleus of the optic tract, the anterior pretectal nucleus, and the posterior pretectal nucleus (Figure 3D,E). Sparsely innervated pretectal regions included the nucleus of the posterior commissure and the olivary pretectal nucleus (Figure 3D,E). SC vGAT+ projections also diffusely innervated the lateral habenula ipsilaterally (Figure 3A,B). In the caudal hypothalamus, SC GABAergic neurons sent a strong projection to the posterior hypothalamus ipsilaterally and a weaker projection contralaterally (Figure 3A,C). vGAT+ projections also weakly innervated the subthalamic nucleus (Figure 3A,B).

**FIGURE 3:**
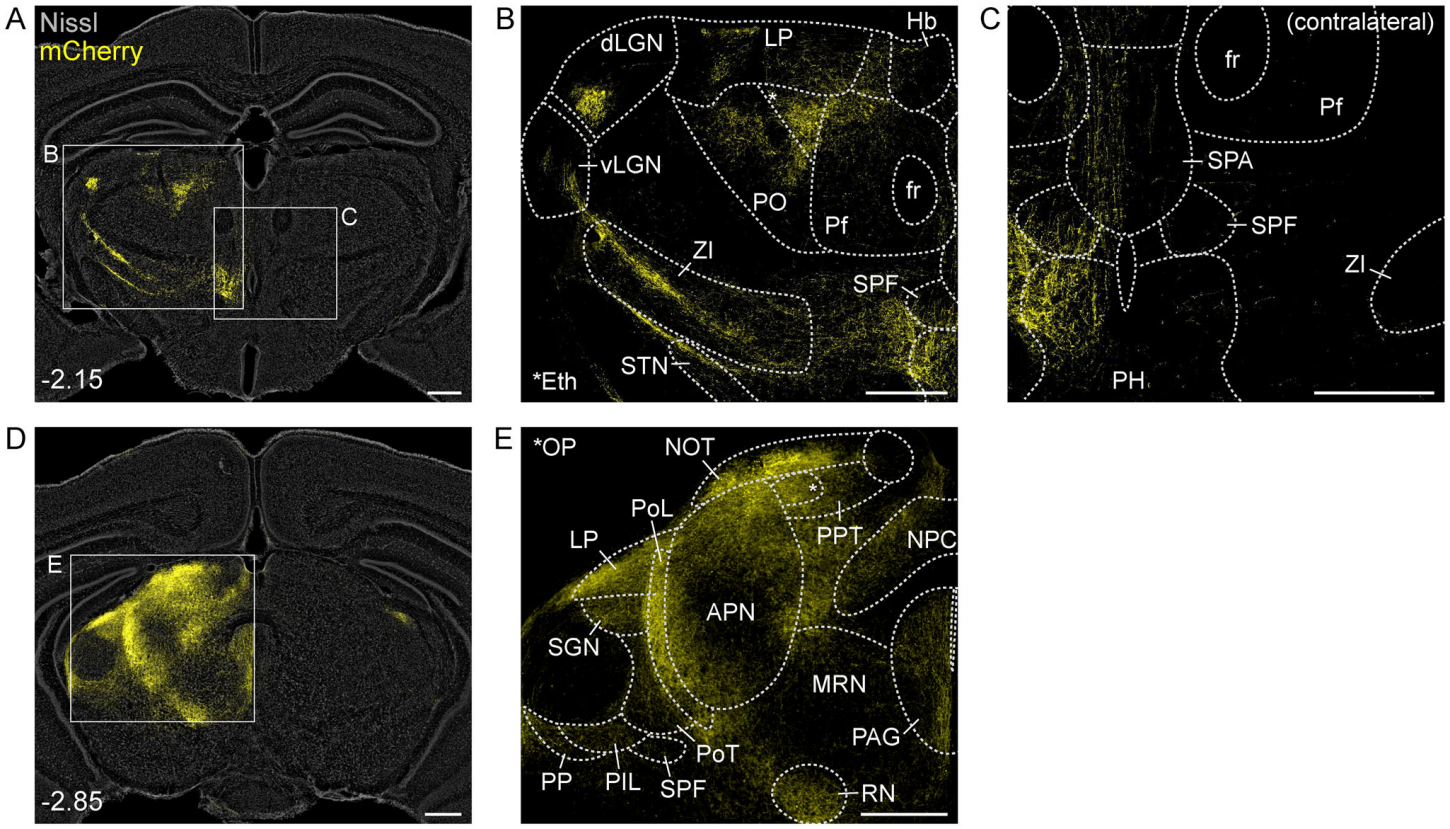
SC inhibitory neurons project to nuclei in the caudal diencephalon. (A–E) SC vGAT+ (yellow) axonal innervation in caudal diencephalon coronal sections with Nissl counterstain (gray) (left column) and magnified regions of interest (center and right columns) from the same case presented in Figure 2. Scale bars = 500 µm.

Inhibitory SC vGAT+ neurons projected to the midbrain and hindbrain (Figures 3 and 4), with strong innervation observed in the red nucleus (Figure 3D,E), pontine gray (Figure 4A,B), and periaqueductal gray (particularly the lateral subregion in central and posterior sections) (Figure 4C,D,I,J), in addition to the parabigeminal, sagulum, and midbrain reticular nuclei (Figure 3C,D). Strong ipsilateral innervation was also observed in the pedunculopontine and rostral pontine reticular nuclei (Figure 4F,G), as well as the inferior colliculus (notably the external cortex) and cuneiform nuclei (Figure 4I,J). In tissue sections containing labeled SC vGAT+ cells, we observed sparse innervation of the contralateral superior colliculus (Figure 4F,H). Inhibitory axons were also sparsely present in the contralateral periaqueductal gray (Figure 4F,H,I,J). Ipsilateral hindbrain efferents sparsely targeted by the vGAT+ projection included the tegmental reticular (Figure 4C,E) and caudal pontine reticular (Figure 4F,G,I,K) nuclei.

**FIGURE 4:**
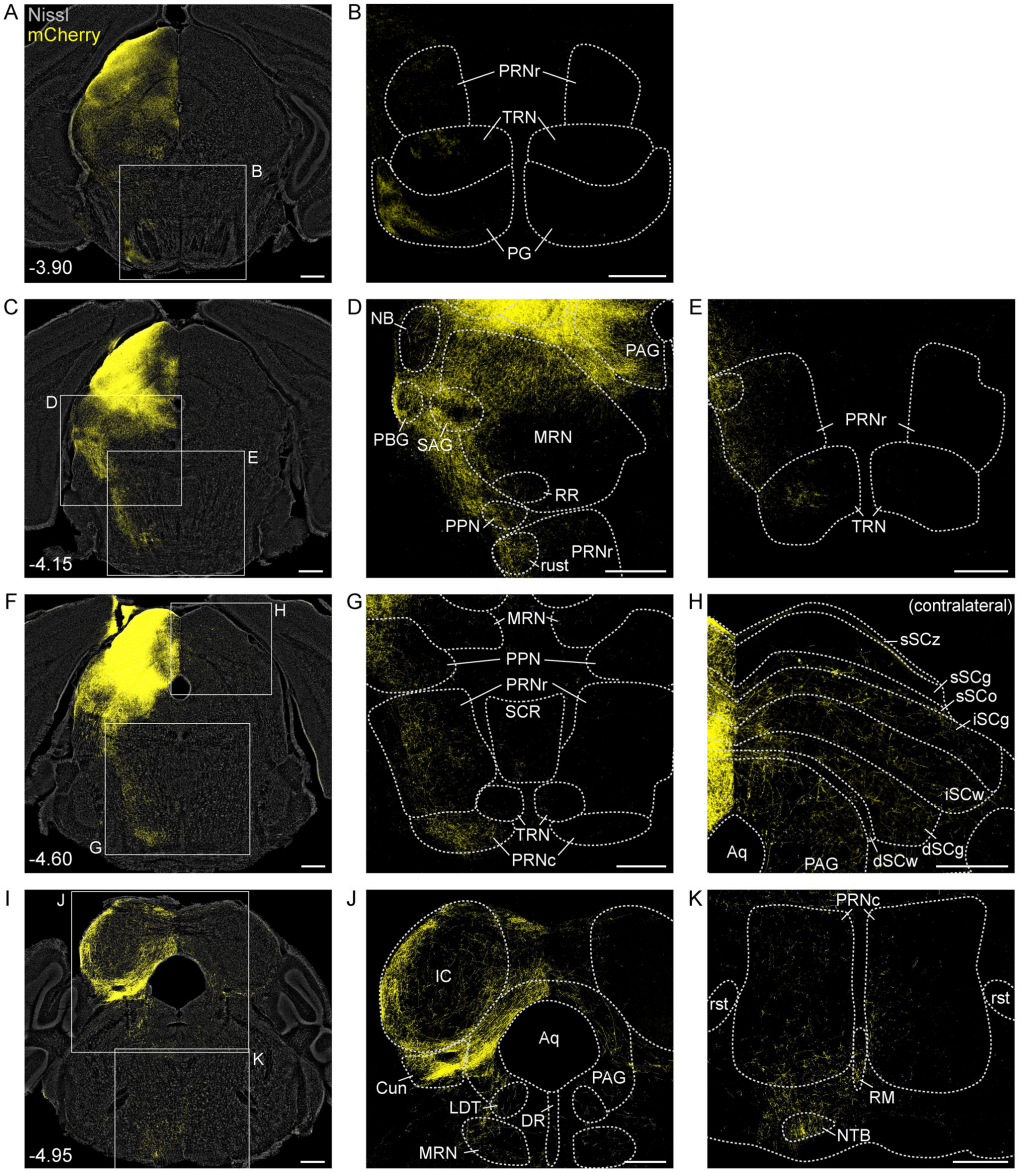
SC inhibitory neurons innervate the midbrain and hindbrain. (A–K) SC vGAT+ (yellow) axonal innervation in midbrain and hindbrain coronal sections with Nissl counterstain (gray) (left column) and magnified regions of interest (center and right columns) from the same case presented in Figure 2. Scale bars = 500 µm.

### 3.2 SC Excitatory Projections

Following the same methodological approach, we used the vGlut2‐Cre reporter mouse line to label glutamatergic SC projections (Figure 5A). We observed a multitude of efferent targets in the rostral diencephalon (Figure 5). In the hypothalamus, excitatory vGlut2+ axons diffusely innervated the lateral and anterior hypothalamic nuclei (Figure 5B,C) as well as the subthalamic nucleus (Figure 5G,I). Excitatory axons strongly innervated ipsilateral central lateral (Figure 5D,E), lateral posterior (Figures 5G,H and 6A,B), and ventromedial (Figure 5D,E,G,I) thalamic nuclei. Weaker ipsilateral innervation was detected in the lateral mediodorsal, paracentral, and central medial nuclei (Figure 5D,E). Weak ipsilateral innervation was also observed in the zona incerta (Figures 5G,I and 6A,B). SC excitatory projections also extended contralaterally, weakly innervating the central lateral and ventromedial thalamic nuclei (Figure 5D,F).

**FIGURE 5:**
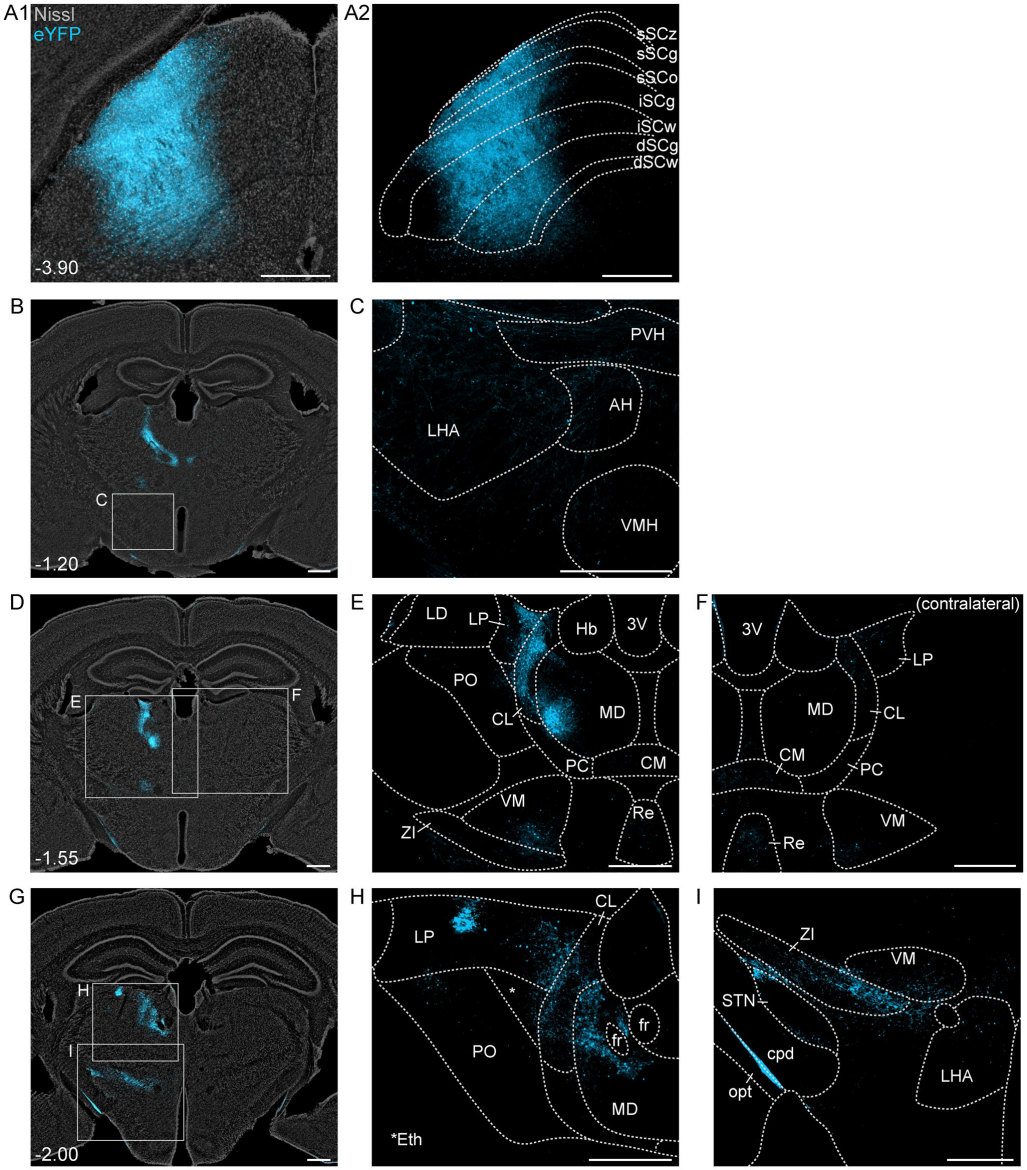
SC excitatory neurons innervate nuclei in the anterior diencephalon. (A) vGlut2+ cells expressing eYFP fluorophore (blue) in the SC of a representative case (#906) with Nissl counterstain (gray). A Cre‐dependent fluorophore‐expressing anterograde virus was unilaterally injected into the SC of vGlut2‐Cre transgenic mice. (B–I) SC vGlut2+ axonal innervation of nuclei, with coronal sections shown in the left column and magnified regions of interest in the center and right columns. Scale bars = 500 µm.

**FIGURE 6:**
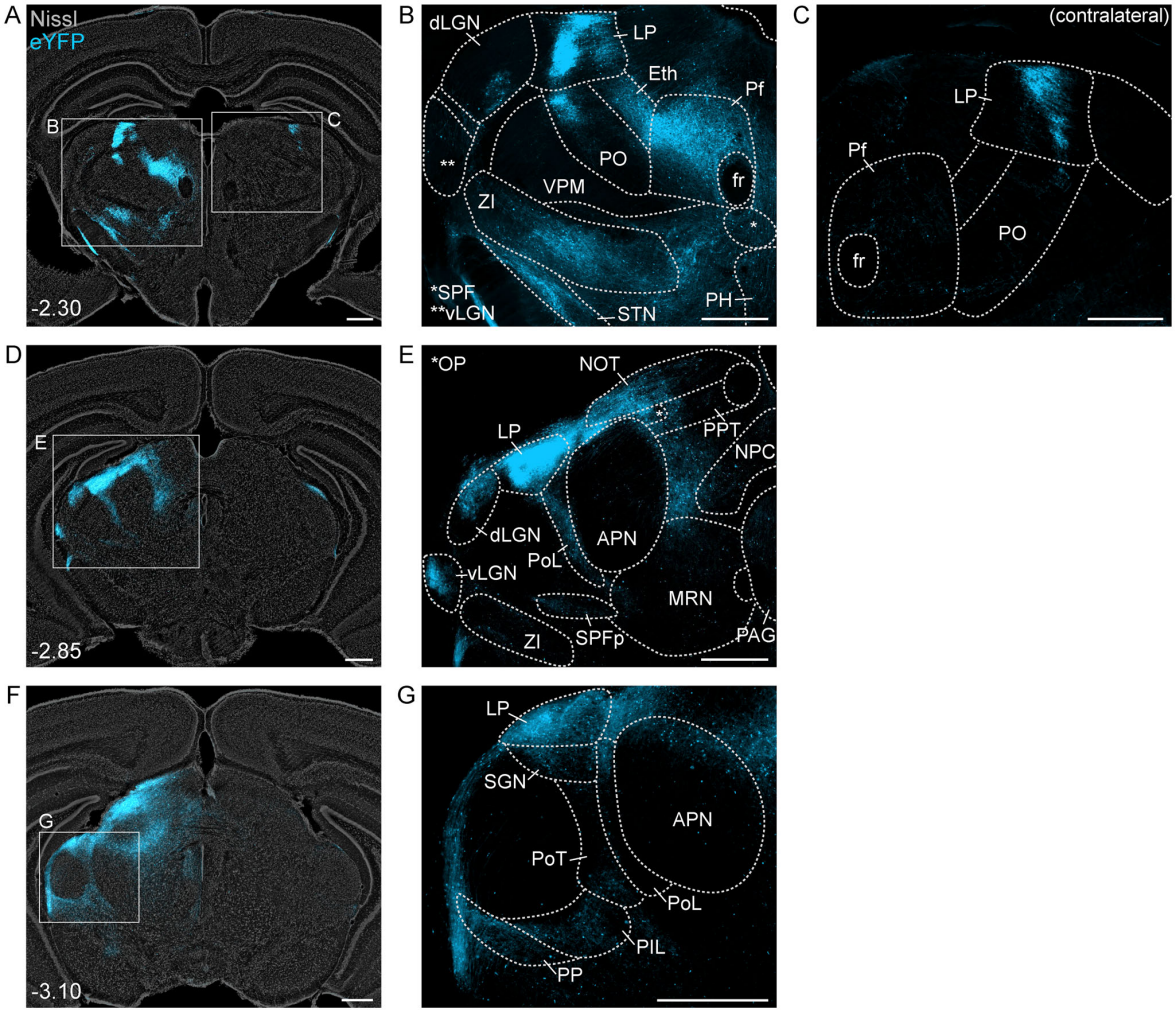
SC excitatory neurons project to the caudal diencephalon. (A–G) SC vGlut2+ (blue) innervation in caudal diencephalon coronal planes with Nissl counterstain (gray) in the left column and magnified regions of interest in the center and right column micrographs from the same case shown in Figure 5. Scale bars = 500 µm.

Excitatory vGlut2+ axons projected to a range of efferents in the caudal diencephalon (Figure 6). In the thalamus, SC projections strongly innervated ipsilateral lateral posterior, parafascicular, and ethmoid nuclei (Figure 6A,B), as well as the dorsal lateral geniculate nucleus (Figure 6D,E). Weaker ipsilateral innervation was detected in the posterior complex and ventral posterior complex of the thalamus (Figure 6A,B). Other weakly innervated caudal thalamic nuclei included the ipsilateral ventral lateral geniculate, suprageniculate, subparafascicular, posterior limiting, posterior triangular, and peripeduncular nuclei (Figure 6D,E,F,G). Weak ipsilateral innervation was also observed in the posterior hypothalamic nucleus (Figure 6A,B). Weakly targeted contralateral efferents included the parafascicular and lateral posterior thalamic nuclei (Figure 6A,C). Within the pretectum, SC excitatory neurons strongly innervated the nucleus of the optic tract and posterior pretectal nucleus and more weakly projected to the nucleus of the posterior commissure, the anterior pretectal nucleus, and the olivary pretectal nucleus (Figure 6D–G).

Glutamatergic SC neurons innervated nuclei in the midbrain and hindbrain (Figure 7). vGlut2+ axons strongly innervated the ipsilateral parabigeminal and sagulum nuclei (Figure 7G,H), as well as the pontine gray (Figure 7D,F), midbrain reticular nucleus (Figure 7D,E,G,H), and cuneiform nucleus (Figure 7J,K). SC excitatory projections weakly projected ipsilaterally to central and posterior sections of the periaqueductal gray, particularly the lateral subregion (Figure 7G,H). We also observed extremely weak ipsilateral innervation of the external cortex of the inferior colliculus (Figure 7J,K). In sections containing labeled SC vGlut2+ cell bodies, we observed weak axonal innervation of the contralateral periaqueductal gray and superior colliculus (Figure 7A,C). Excitatory SC neurons sent a strong and exclusively contralateral projection to the tegmental reticular nucleus (Figure 7G,I). We also found that the vGlut2+ projection bilaterally innervated the pedunculopontine nucleus and pontine reticular nucleus (both rostral and caudal subregions) (Figure 7G,I,J,L). Within these nuclei, the contralateral projection was largely confined to medial regions, whereas the ipsilateral projection targeted lateral areas.

**FIGURE 7:**
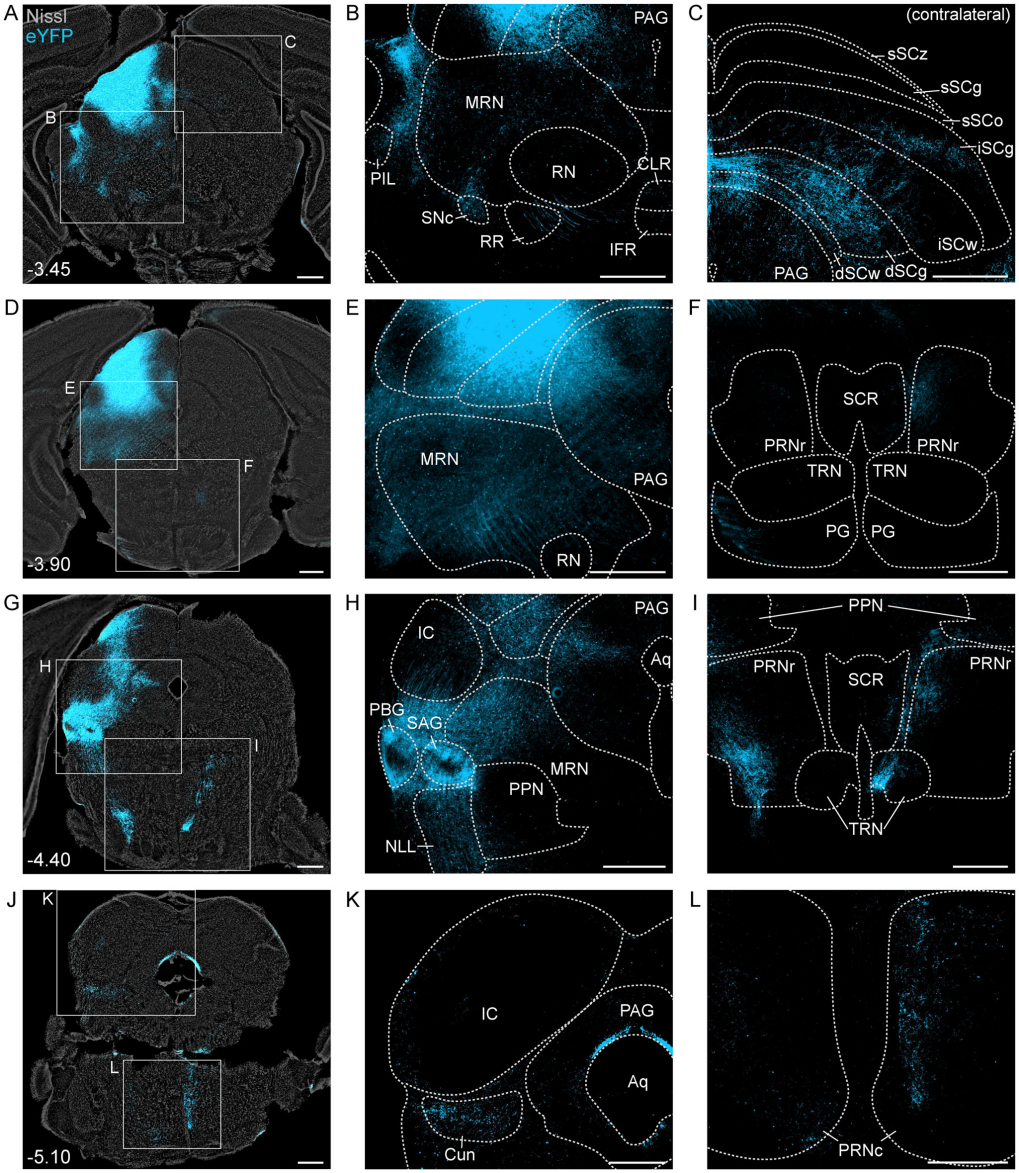
SC excitatory neurons project to the midbrain and hindbrain. (A–L) SC vGlut2+ (blue) innervation in midbrain and hindbrain coronal planes with Nissl counterstain (gray) in the left column and magnified regions of interest in the center and right column micrographs from the same case shown in Figure 5. Scale bars = 500 µm.

### 3.3 Comparison of SC Inhibitory and Excitatory Projections

SC projection neuron tracing in vGAT‐Cre and vGlut2‐Cre reporter lines revealed a range of shared and exclusive efferent targets. However, it is challenging to precisely compare SC innervation patterns based on labeled neurons in different mice. To directly examine efferent targeting by the two neuronal populations in the same brain, we employed a Cre on/off dual‐labeling strategy. Two fluorophore‐expressing viruses were co‐injected into the SC of vGAT‐Cre mice: one virus expressed tdTomato in all Cre‐expressing (vGAT+) cells and the other virus expressed eYFP in all cells not expressing Cre (VGAT−). This approach allowed us to label both inhibitory and putative excitatory SC projection neurons in the same brain (Figure 8A).

**FIGURE 8:**
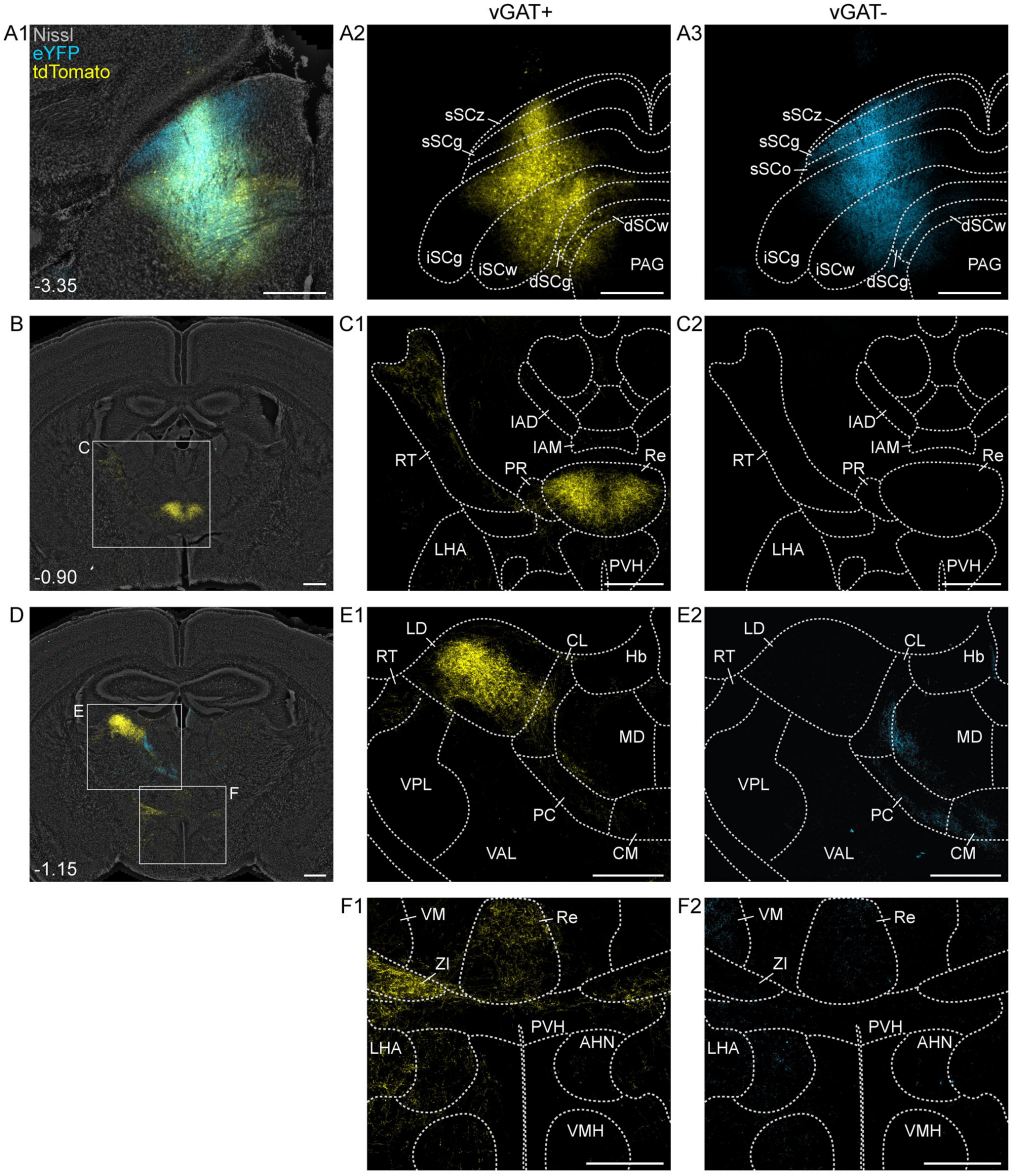
Inhibitory and putative excitatory SC neurons differently innervate the anterior diencephalon. (A) vGAT+ cells expressing tdTomato (yellow) and vGAT− cells expressing eYFP (blue) in the SC of a representative case (#1692) with Nissl counterstain (gray). A pair of Cre‐on and Cre‐off viruses was unilaterally injected into the SC of vGAT‐Cre transgenic mice to selectively label cells with different fluorophores based on Cre recombinase expression. (B–F) SC vGAT+ and vGAT− axonal innervation of thalamic and hypothalamic nuclei, with coronal sections shown in the left column and magnified regions of interest in the center (vGAT+) and right (vGAT−) columns. Scale bars = 500 µm.

Axonal patterns produced by the dual‐tracer approach (Figures 8, 9, 10, 11) are consistent with our reported findings from the single‐tracer cases. In the anterior thalamus, we found that vGAT+ cells exclusively innervated the ipsilateral reticular (Figure 8B,C) and lateral dorsal (Figure 8D,E) thalamic nuclei. Whereas vGAT+ cells strongly projected to the entirety of the nucleus of the reuniens, putative excitatory vGAT− cells weakly targeted only the most caudal section of this nucleus (Figure 8B,C,D,F). In contrast, vGAT− cells, but not vGAT+ cells, strongly innervated the paracentral, central medial, and ventral medial nuclei (Figure 8D–F).

**FIGURE 9:**
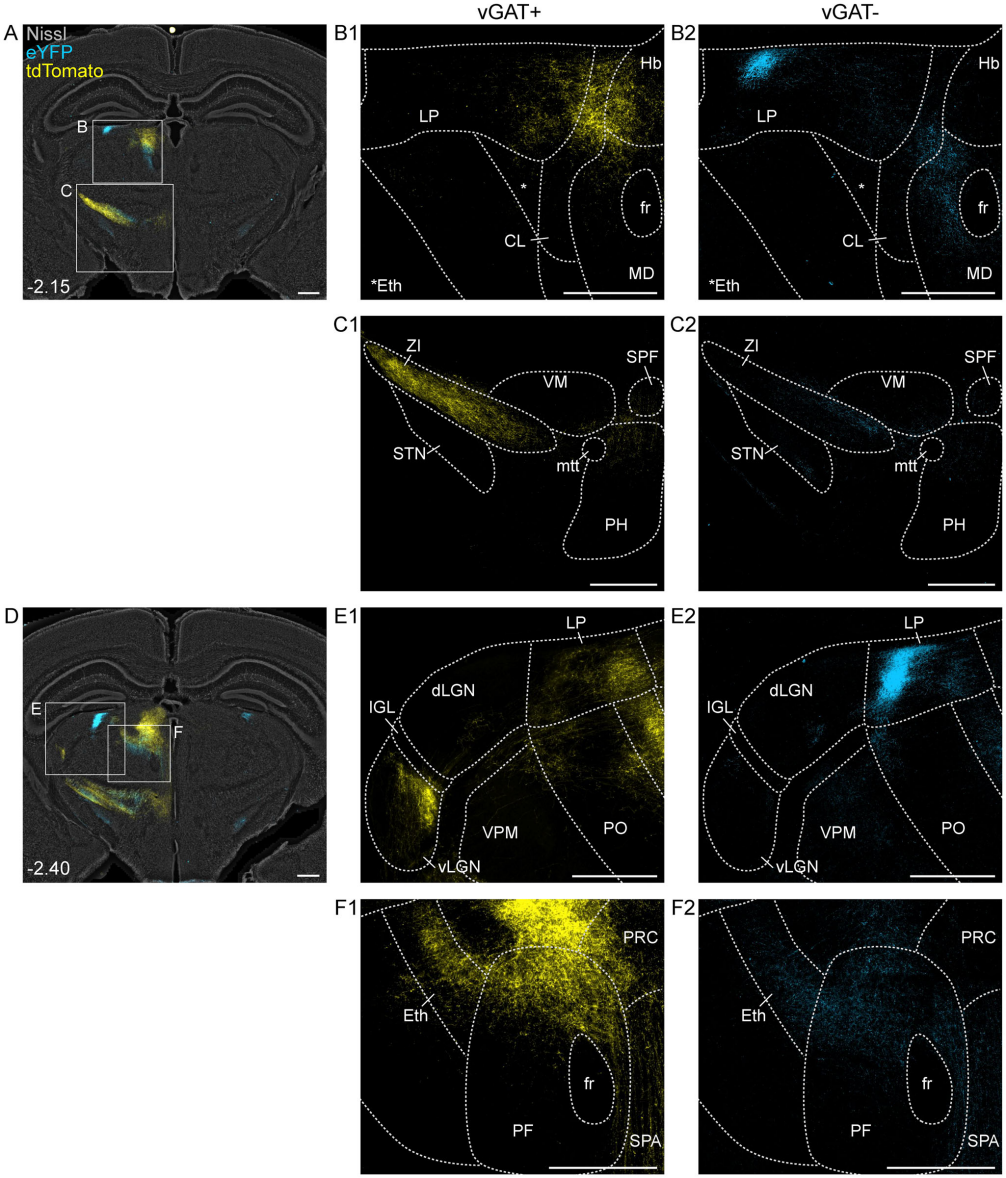
Innervation patterns of SC inhibitory and putative excitatory neurons in posterior thalamus and hypothalamus. (A–F) SC vGAT+ (yellow) and vGAT− (blue) innervation of coronal sections with Nissl counterstain (gray) in the left column and magnified efferent targets in the center (vGAT+) and right (vGAT−) column micrographs from the same case shown in Figure 8. Scale bars = 500 µm.

**FIGURE 10:**
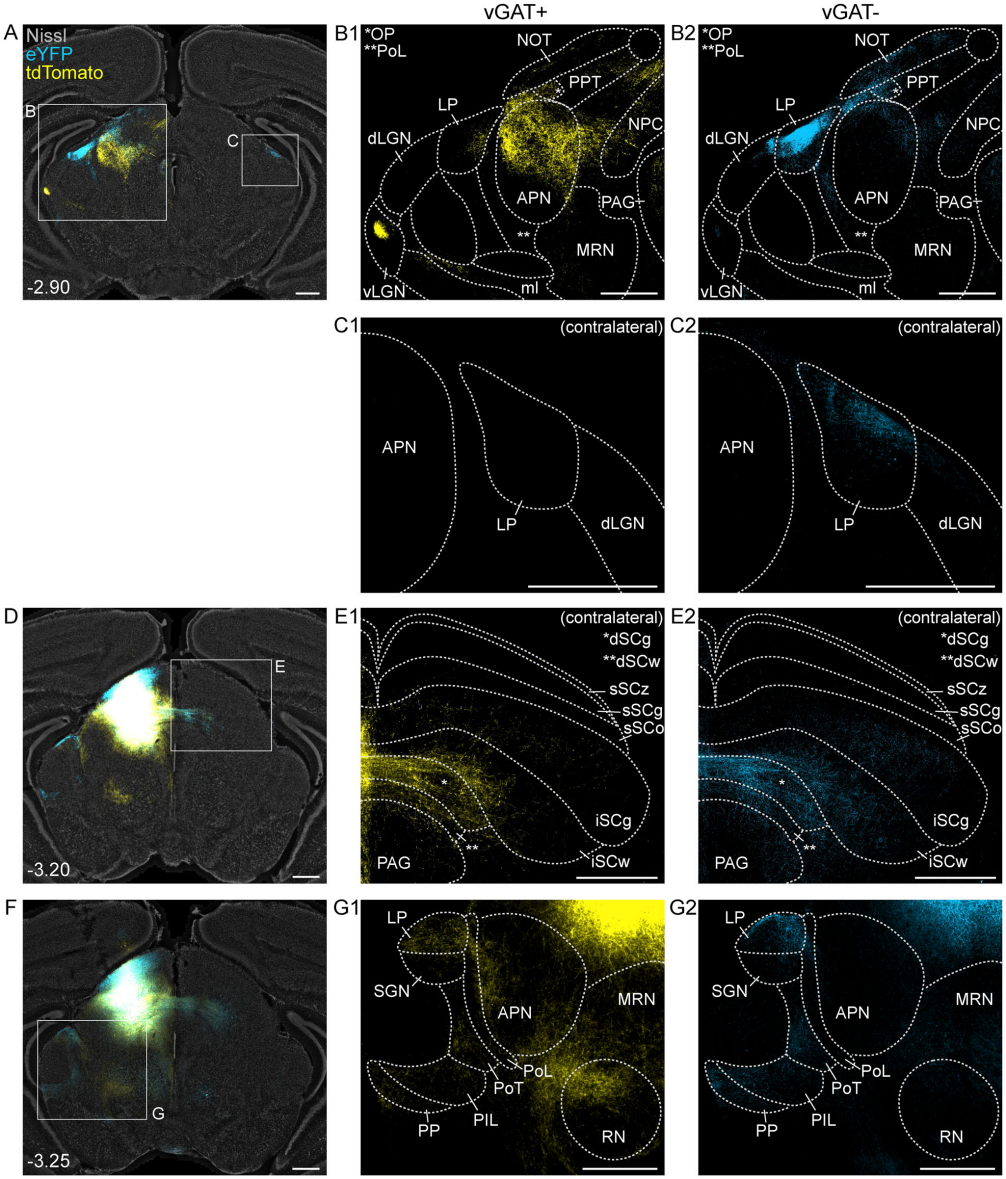
SC inhibitory and putative excitatory neurons have shared and different efferent targets in the caudal diencephalon and midbrain. (A–G) SC vGAT+ (yellow) and vGAT− (blue) axons in coronal sections with Nissl counterstain (gray) in the left column and magnified regions of interest in the center (vGAT+) and right (vGAT−) column micrographs from the same case shown in Figure 8. Scale bars = 500 µm.

**FIGURE 11:**
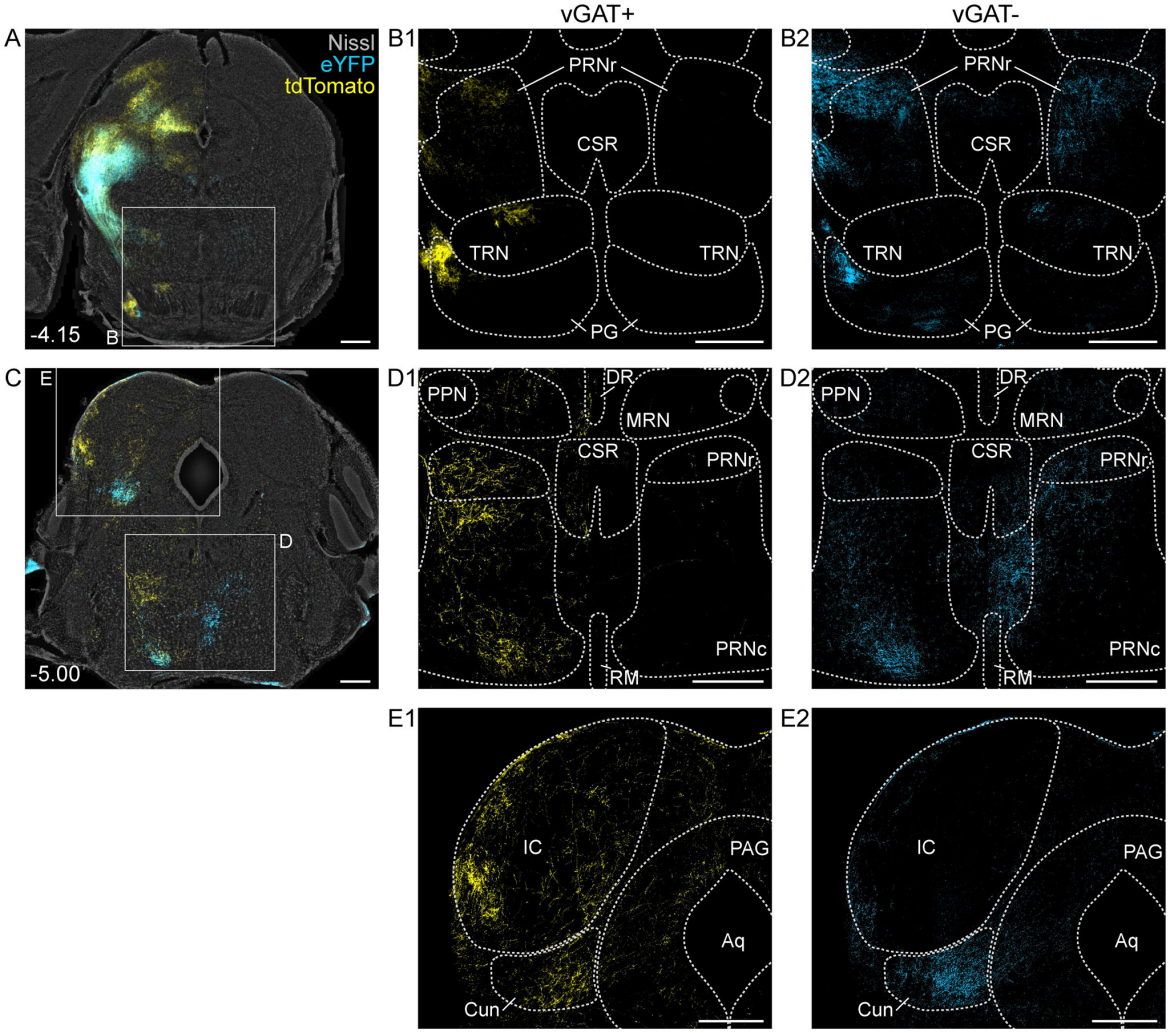
SC inhibitory and putative excitatory projections in the midbrain and hindbrain. (A–E) SC vGAT+ (yellow) and vGAT− (blue) axons in coronal sections with Nissl counterstain (gray) in the left column and magnified regions of interest in the center (vGAT+) and right (vGAT−) column micrographs from the same case shown in Figure 8. Scale bars = 500 µm.

More posterior diencephalon sections revealed distinct innervation patterns from the two SC neuronal populations (Figure 9). In the lateral posterior thalamic nucleus, vGAT+ cells preferentially targeted medial areas, with diffuse innervation spreading laterally (Figure 9A,B,D,E). In contrast, vGAT− cells sent a highly localized projection to the lateral region of this nucleus. Furthermore, the vGAT− projection to the lateral posterior nucleus appeared relatively denser in more posterior sections, which was not observed for vGAT+ labeled fibers (Figure 10A,B). vGAT−, but not VGAT+, SC cells also sent a contralateral projection to this thalamic nucleus (Figure 10A,C). We also found differences in the innervation of the zona incerta. vGAT+ cells innervated the entire rostral–caudal axis of this nucleus and targeted the ventral tier more strongly. Conversely, putative excitatory vGAT− cells tended to innervate the dorsal medial subregion of the zona incerta in more caudal sections (Figures 8D,F and 9A,C).

Within the pretectal area, excitatory and inhibitory SC projections generally showed similar innervation patterns (Figure 10A,B,F,G). However, we observed a notable difference in the anterior pretectal nucleus. vGAT+ SC cells sent a strong projection that innervated the majority of the nucleus but was densest dorsally. In contrast, the vGAT− projection diffusely targeted a limited portion of the nucleus along the dorsal boundary. We also observed that only vGAT+ cells projected to the red nucleus of the midbrain (Figure 10F,G). Both populations innervated the contralateral SC; however, we found vGAT− axons to predominantly innervate more lateral subregions of the intermediate layers, whereas vGAT+ terminals were also present in the medial intermediate layers (Figure 10D,E).

In the pons, the two SC populations showed both convergent and divergent innervation patterns (Figure 11). In the rostral pontine reticular nucleus, both projections innervated similar ipsilateral subregions; however, vGAT− terminals additionally innervated the nucleus contralaterally (preferentially in the more medial areas) (Figure 11A,B). A similar pattern was observed in the caudal pontine reticular nucleus (Figure 11C,D). We found that both SC populations focally innervated a lateral subregion of the ipsilateral pontine gray and that vGAT− cells provided an additional and more diffuse input to more medial areas of this nucleus (Figure 11A,B). The tegmental reticular nucleus was innervated ipsilaterally by vGAT+ projections and contralaterally by vGAT− terminals (Figure 11A,B). Lastly, we observed that vGAT+ cells innervated the entirety of the inferior colliculus with a particularly strong projection to the external cortex (Figure 11C,E). In contrast, this nucleus was largely devoid of input from vGAT− cells, except for very weak terminations in the lateral edge of the external cortex.

In summary, the use of the Cre on/off dual‐labeling strategy is consistent with the overall patterns of innervation we found using vGAT‐Cre and vGlut2‐Cre reporter lines. More importantly, this approach verified that differences in the innervation patterns between inhibitory and putative excitatory projections can be observed from the same region of the SC.

## 4 Discussion

The present study identified distinct efferent innervation patterns of SC GABAergic and glutamatergic projection neurons (quantified for each case in Figures 12 and 13 and summarized in Figure 14). Some targets were solely innervated by one population. The lateral dorsal and reticular thalamic nuclei, lateral habenula, and red nucleus were targeted exclusively by inhibitory vGAT+ cells. In contrast, excitatory vGlut2+ SC cells solely innervated the central medial intralaminar thalamus. Other efferents were unequally targeted by the two populations. The inferior colliculus and zona incerta, as well as reuniens, anterior pretectal, and posterior hypothalamic nuclei, received dense input from vGAT+ cells and weak or confined innervation from vGlut2+ cells. Conversely, the parafascicular intralaminar and ventral medial thalamic nuclei received strong input from vGlut2+ but not vGAT+ neurons. We also found that the SC vGlut2+ population more often sent cross‐hemispheric projections to efferents in the thalamus and pons. Projections to shared efferent targets often showed disparate expression patterns. vGAT+ and vGlut2+ neurons innervated different subregions of the zona incerta and lateral posterior thalamus, for example. Together, these results demonstrate that inhibitory long‐range projections are a major and unique component of SC efferent connectivity.

**FIGURE 12:**
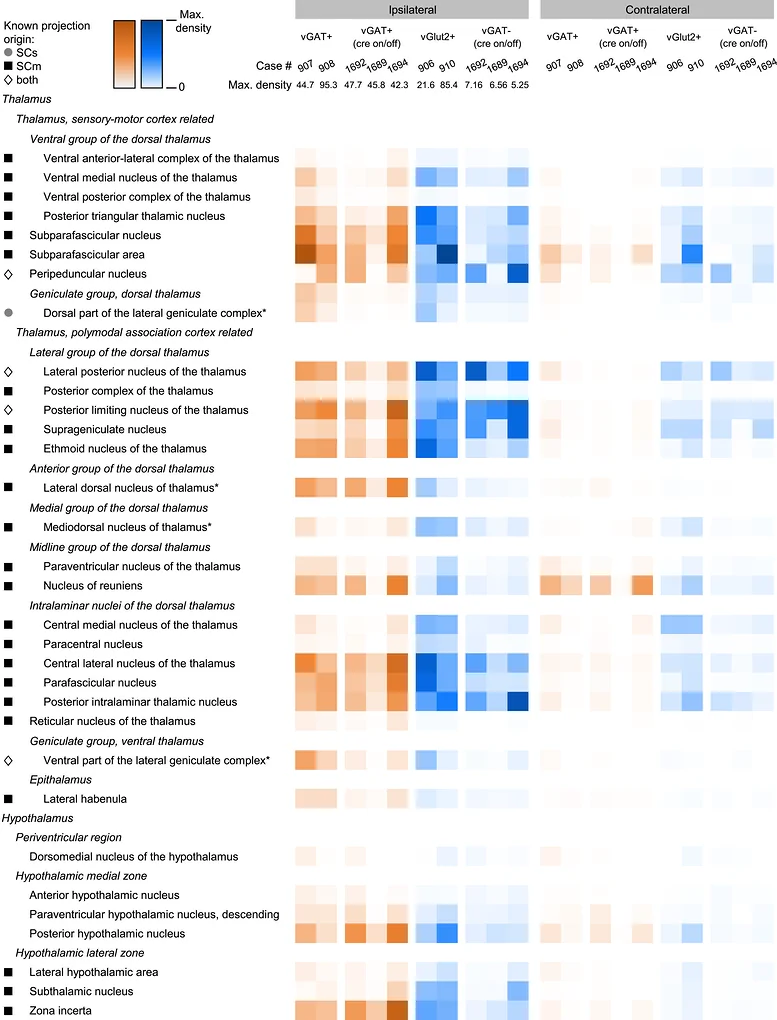
SC projection innervation of diencephalon targets. Innervation density in thalamic and hypothalamic nuclei represented by heat maps from dark (high density) to light (low density) colors for each vGAT+ (orange) and vGlut2+ or vGAT− (blue) projection. Each case is normalized to the densest target for that case. The maximum density for each case is listed below the case number (axon terminal counts per cubic meter). Efferents are separately shown for the hemisphere ipsilateral (left) and contralateral (right) to the injected SC. Asterisks indicate nuclei in which innervation was confined to a subregion, limiting the interpretation of density values. Known SC layer origins for projections are indicated by gray circles (superficial layers), black squares (deeper layers), or white diamonds (both) (Cai et al. 2019; Cang et al. 2024; Foster et al. 1989; Liu et al. 2022; May 2006; Whyland et al. 2020; Zahm et al. 2011; Zhang et al. 2019; Zingg et al. 2017). See also Figure 13.

**FIGURE 13:**
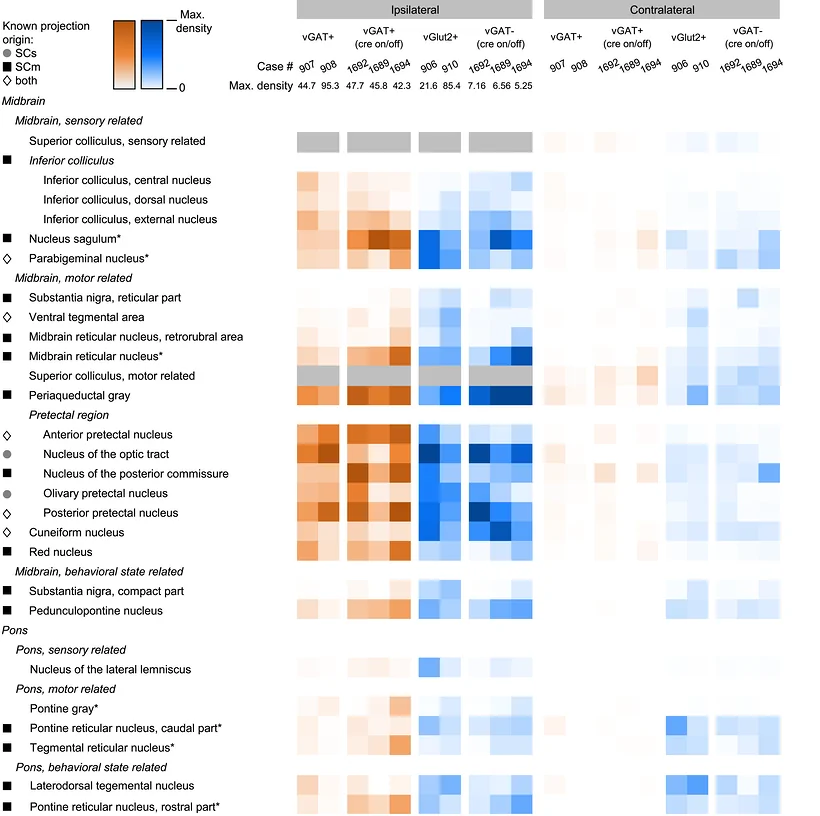
SC projection innervation of midbrain and hindbrain targets. Same as Figure 12, but for efferent targets in midbrain and hindbrain nuclei. Color gradient ranges for the cases are the same as in Figure 12. Axonal densities from ipsilateral superior colliculus layers (gray boxes) were excluded from analysis. Asterisks indicate nuclei in which innervation was confined to a subregion, limiting the interpretation of density values. Known SC layer origins for projections are indicated by gray circles (superficial layers), black squares (deeper layers), or white diamonds (both).

**FIGURE 14:**
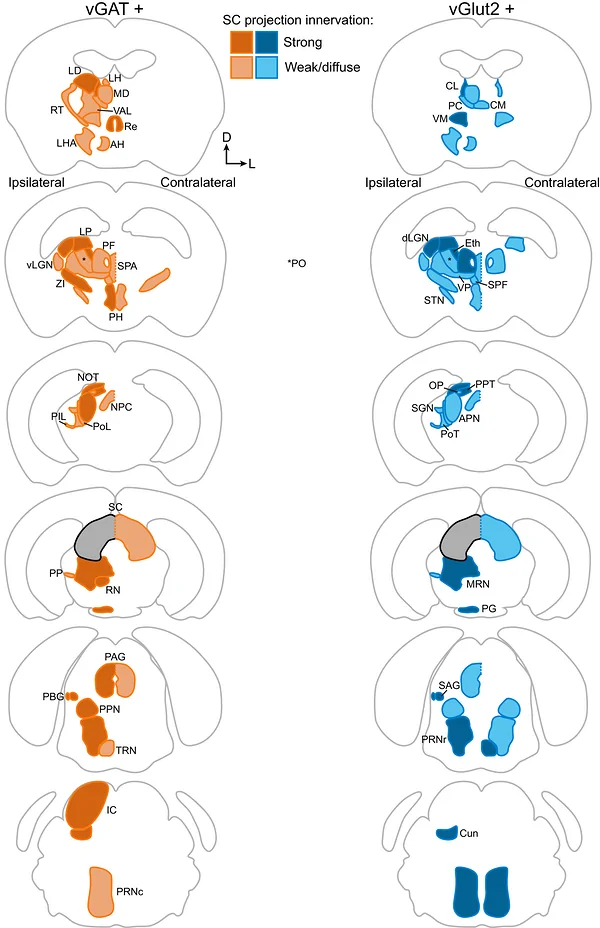
Summary of SC inhibitory and excitatory projection efferents. Ipsilateral and contralateral targets of vGAT+ (left column, orange) and vGlut2+ (right column, blue) SC neurons in coronal atlas schematics from anterior (top) to posterior (bottom) planes. Innervation density (assessed within‐case) is denoted by darker fill shades for dense labeling (in either the entire nucleus or a subregion) and lighter fill shades for weak or diffuse innervation (in either the entire nucleus or a subregion). Schematics summarize significant and consistent axonal expression observed across all cases. For visual simplicity, subregional innervation patterns are not depicted.

### 4.1 Superior Collicular Inhibitory Neurons

We utilized the vGAT reporter line to label all inhibitory neurons in the mouse SC. Other studies characterizing inhibitory projections from the superficial SC employed GAD67 or GAD2 transgenic lines. Examination in the superficial gray layer reveals that only 60% of GABAergic cells express GAD67 and are most likely interneurons (Whyland et al. 2020). In contrast, GAD2+ superficial SC cells are shown to project to the lateral geniculate and parabigeminal nuclei, whereas cells located in ventral layers are found to innervate the pretectum (Gale and Murphy 2014; Li et al. 2023; Whyland et al. 2020). GAD2+ likely encompasses distinct neuronal groups. GAD2+ projections from superficial SC to the lateral geniculate and parabigeminal arise from distinct populations that receive unique afferent inputs (Li et al. 2023).

Similarly, vGAT is expressed by a heterogeneous collection of GABAergic cell types that vary in morphology and molecular markers. Whereas inhibitory neurons are traditionally recognized by a horizontal somatodendritic profile (Gale and Murphy 2014; May 2006), SC GABAergic neurons exhibit a range of morphological properties. Horizontal cells account for only 25% of GAD2‐expressing cells in the superficial gray layer (Whyland et al. 2020) and 10% of GAD67‐expressing cells in the intermediate SC (Sooksawate et al. 2011). Single‐cell sequencing techniques further illustrate the diversity of inhibitory neuronal groups in the SC by identifying populations with unique RNA expression profiles (Xie 2021; Choi 2023; Chen et al. 2025). Use of transgenic mouse lines selective for GABAergic subtypes discovered from these analyses may reveal selective efferent pathways that are otherwise masked in vGAT or GAD reporter lines. For example, Pax7‐expressing GABAergic cells located in the superficial SC solely innervate the ventral lateral geniculate nucleus (Choi et al. 2023). Evaluating molecularly defined subtypes (for both inhibitory and excitatory populations) may further elucidate the organization of SC efferent projections and aid in identifying specific behavioral roles.

### 4.2 Long‐Range Inhibitory Functional Motifs

How may long‐range inhibition facilitate collicular‐mediated function and behavior? Some recent studies have demonstrated that SC GABAergic projections are behaviorally relevant. Discrete populations of GAD2+ cells in the superficial SC innervate the lateral geniculate and parabigeminal nuclei; these pathways promote freezing and escape responses to visual looming, respectively (Li et al. 2023). Given the SC's established role in ascending and descending visual pathways, inhibitory projections to visual and orienting‐associated regions (i.e., lateral geniculate, lateral posterior, pretectal, and pontine nuclei) may modulate responses to visual stimuli. Additionally, our study revealed a number of seemingly non‐visual‐related nuclei that were predominantly innervated by SC inhibitory projections. The lateral dorsal and reuniens thalamic nuclei, for example, are associated with learning and memory through their connectivity with the limbic system (Perry and Mitchell 2019; Vertes et al. 2022). How an exclusively GABAergic input from the SC to these regions may contribute to limbic function remains to be determined. Similarly, the finding of a strong inhibitory projection to the anterior pretectal nucleus, an area associated with antinociception (Genaro and Prado 2021), suggests the possibility for the SC to modulate pain perception.

In addition to providing direct feedforward inhibition, long‐range GABAergic projections may also facilitate feedforward *dis*inhibition. The thalamus largely lacks inhibitory interneurons and relies on GABAergic afferents for inhibitory control. Known contributors of thalamic inhibition include the zona incerta, thalamic reticular nucleus, anterior pretectal nucleus, and pontine reticular formation (Halassa and Acsády 2016). We found that SC vGAT+ cells innervate these sources of thalamic inhibition, suggesting that the SC is anatomically well‐positioned to disinhibit thalamic nuclei.

Within the SC, inhibitory signaling is provided by both GABAergic interneurons and extrinsic inhibitory inputs from several diencephalic and midbrain nuclei. SC GABAergic projections to these afferents may offer a mechanism for the SC to gate or suppress inhibitory input. Both the ventral lateral geniculate nucleus and ventral zona incerta have reciprocal GABAergic connections with the SC. These collicular afferents are associated with defensive responses to expected visual threat (Fratzl et al. 2021) and orienting behavior (Hormigo et al. 2023). SC inhibition of these nuclei may occlude inhibitory input to itself, effectively providing a means for internally driven disinhibition. Given that the SC is a central node for both ascending and descending visual pathways, such a mechanism may grant the SC a means to alter stimulus–response behavioral contingencies to threat‐ (or reward‐) associated visual cues, as opposed to acting as a passive relay for top‐down motor commands. This reciprocal GABAergic circuit arrangement may also facilitate modulation of neuronal habituation to sensory stimuli. The ventral lateral geniculate nucleus innervates the medial SC, whereas the zona incerta input to the SC is topographically organized along the rostral–caudal axis and inversely along the medial–lateral axis (Mitrofanis 2005). Given that the SC itself is retinotopically organized, it is possible that SC‐driven suppression of these inhibitory inputs may selectively augment collicular responses to different areas of the visual field.

The widespread innervation of the diencephalon, midbrain, and hindbrain by both SC excitatory and inhibitory projections presupposes some manner of coordinated organization between the two neurotransmitter systems. In the hindbrain, we observed a consistent arrangement of ipsilateral innervation by SC vGAT+ cells and contralateral innervation by vGlut2+ cells. These two projections drive complementary orienting movements (Isa et al. 2020; Sooksawate et al. 2013). In other areas, we observed differences in targeting of shared efferents. For example, SC inhibitory cells projected to medial areas of the lateral posterior thalamic nucleus, whereas the excitatory SC projection innervated the nucleus laterally. Although specific behavioral functions have yet to be identified that align with these differences in SC innervation, there is evidence for subregional differences in anatomical connectivity of this nucleus (Bennett et al. 2019; Leow et al. 2022). In many nuclei, however, we observed overlapping innervation patterns by the two projection types. These SC inputs may potentially synapse on distinct populations within a given nucleus. Alternatively, SC inhibitory and excitatory projection neurons may fire differently in a context‐ or state‐dependent manner, providing a mechanism for the SC to bidirectionally modulate efferent activity.

### 4.3 Methodological Considerations and Future Directions

Our surgical approach aimed to label a large population of neurons across the SC layers without spreading into the cortex or other midbrain structures. As a consequence, our study traced projections predominantly originating from the intermediate and deep layers of the central SC (Figure 1; Table 2). Some efferents are targeted by projection neurons residing exclusively in more medial or lateral areas of the SC, which may not be represented in our cases. For example, we observed weak and inconsistent axonal labeling in the nucleus of the lateral lemniscus and the paraventricular thalamic nucleus, regions that are innervated by the medial SC (Benavidez et al. 2021). Similarly, we only observed extremely weak innervation of the ventral tegmental area and substantia nigra, which may arise from projection neurons arising from the medial or superficial layers of the SC (Solié et al. 2022; Zhang et al. 2019). Variations in injection targeting combined with smaller sample sizes present challenges in interpreting the innervation patterns we observed in the present study. Future studies employing retrograde tracing in an efferent of interest may serve to validate and further characterize SC projection connectivity.

Prior tracing studies describe the collicular projection to the dorsal lateral geniculate nucleus as terminating in the shell subregion (Bickford et al. 2015). Whereas we observed similar innervation patterns from both vGAT+ and vGlut2+ populations in posterior sections, we also found that both projections descended ventromedially to target the core subregion in anterior sections. Although this is not the first report of such a finding (Gale and Murphy 2014), future work is needed to validate the source of this core‐targeting input.

The superior colliculus layers are organized such that retinal signals and visual cortical inputs are relayed through the superficial layers, whereas the deeper layers integrate multimodal sensory signals and participate in descending motor pathways (May 2006). Identifying which layers contribute to a given efferent projection will aid in elucidating behavioral function. This may be accomplished in future studies employing layer‐specific reporter lines.

Cre on/off viral vectors allow for direct comparison of dual populations in the same tissue and are ideal for nuclei composed of two molecularly different neuronal groups, such as the SC (Chen et al. 2025). This approach allowed us to confirm the distinct subregional innervation patterns of vGAT+ and vGlut2+ projections in the zona incerta and lateral posterior thalamic nucleus. However, the efficiency of Cre recombinase expression in transgenic lines is not complete. Caution is therefore necessary in interpreting the results of the vGAT−‐labeled projection, as this population likely included some proportion of GABAergic cells that failed to express Cre. “Cre off” viral transfection is also less robust, resulting in unequal transfected population sizes. For these reasons, our use of Cre on/off viral constructs was to supplement and verify the results we obtained using traditional single‐population labeling approaches.

Our technical approach does not yield insight into the prevalence of collateralization among efferent targets. Do the GABAergic SC neurons that project to the zona incerta, for example, also innervate the thalamus or midbrain? Determining this aspect of efferent organization will inform which circuits may be jointly recruited to facilitate specific behavioral functions. SC neurons that projected to the contralateral pontine reticular formation (which are likely glutamatergic based on our study's findings) were found to also innervate the intralaminar thalamus in addition to other diencephalic and midbrain targets (Isa et al. 2020). Labeling approaches using retrograde adeno‐associated or pseudorabies viruses can aid in identifying collateral targets of specific collicular projections.

Our findings demonstrate that GABAergic neurons are a major source of collicular efferent connectivity in mice. It is uncertain whether this feature is present in other mammalian species. One group identified putatively inhibitory horizontal cells within a population of SC neurons that projected to the lateral posterior thalamic nucleus in the cat (Caldwell and Mize 1981). Anatomical tracing studies in higher‐order species such as ferrets or primates will help determine whether long‐range inhibitory projections are an evolutionarily conserved element of collicular connectivity.

## 5 Conclusion

Long‐range inhibitory projection neurons are a major contributor to superior collicular efferent connectivity in mice. vGAT+ SC neurons innervated a range of nuclei throughout the diencephalon, midbrain, and hindbrain. Comparison of this projection with that of glutamatergic vGlut2+ neurons revealed a complex arrangement of innervation patterns. Whereas some efferents were disproportionately targeted by one projection or exhibited differences in subregional localization, other nuclei showed similar innervation patterns. Both SC populations also demonstrated unique cross‐hemispheric projections. Future work is necessary to delineate the contributions of these two populations and their projection patterns to particular neural functions and behavioral outputs.

## Author Contributions

L.W. and R.J.K. conceived the study. L.W. and K.K.C. conducted the surgeries. K.K.C., R.P., and C.R.G. performed histological processing and analysis. K.K.C. and R.J.K. wrote the paper.

## Conflicts of Interest

The authors declare no conflicts of interest.

## Data Availability

The data that support the findings of this study are available from the corresponding authors upon reasonable request.
